# Modified Atmosphere Systems and Shelf Life Extension of Fish and Fishery Products

**DOI:** 10.3390/foods5030048

**Published:** 2016-06-28

**Authors:** Christina A. Mireles DeWitt, Alexandra C.M. Oliveira

**Affiliations:** 1 OSU Seafood Research & Education Center Experiment Station, Department of Food Science and Technology, Oregon State University, Astoria, OR 97103, USA; christina.dewitt@oregonstate.edu; 2 BluWrap, 766 Harrison Street #102, San Francisco, CA 94107, USA; 3 Kodiak Seafood and Marine Science Center, School of Fisheries and Ocean Sciences, University of Alaska Fairbanks, 118 Trident Way, Kodiak, AK 99615, USA

**Keywords:** modified atmosphere packaging, controlled atmosphere systems, fish and fishery products, seafood shelf life, fish, fishery products, seafood

## Abstract

This review aims at summarizing the findings of studies published over the past 15 years on the application of modified atmosphere (MA) systems for shelf life extension of fish and fishery products. This review highlights the importance of CO_2_ in the preservation of seafood products, and underscores the benefits of combining MA technology with product storage in the superchilled temperature range. It is generally accepted that MA technology cannot improve product quality and should not be utilized as a substitute for good sanitation and strict temperature control. Benefits derived from application of MA, however, can significantly impact preservation of product quality and it subsequent shelf-life. For this reason, this review is the first of its kind to propose detailed handling and quality guidelines for fresh fish to realize the maximum benefit of MA technology.

## 1. Introduction

Modified atmosphere packaging is a technology that dates back to the 1930’s [1] and has been a critical area of research in terms of minimizing waste through spoilage in fishery products. Fish post-harvest losses were estimated in 2015 at 10 to 12 million tons, accounting for approximately 10% of global capture and cultured fish [2]. Microbial spoilage is a major contributor to quality loss and subsequent spoilage of fishery products [3,4]. Modified atmosphere packaging (MAP) combined with low storage temperatures is an effective technique to achieve shelf life extension of fishery products [5,6,7,8,9].

Advances in the application of MAP to shelf life extension of muscle foods are occurring at a fast pace. This is evident by the large body of scientific literature available not only addressing applications in fishery products but also in beef [10], poultry [11] and pork products [12]. A major driver for further extension of shelf life of fresh muscle foods is the ongoing increase in consumer demand for fresh products. Notwithstanding, the adoption of modified, or controlled, atmosphere systems in fish and fishery products has generally been slower than other meat systems due to concerns about *Clostridium botulinum* type E. Although *Clostridium botulinum* is ubiquitously present in the environment, type E is associated with marine food products and it has the ability to produce toxin in low oxygen atmospheres at refrigeration temperatures. Under optimal conditions, the lowest temperature range for *Clostridium botulinum* type E growth and toxin production is 3.0–3.3 °C [13].

Two major reviews of MAP applied to fish and fishery products have been published since 1990. In these reviews [5,14], the microorganisms associated with fresh fish spoilage in aerobic and anaerobic conditions together with the associated chemical changes were covered in detail. In addition, the history and principles of MAP were also overviewed as well as the research findings on the application of MAP to seafood inclusive until 1999. In 2005, a review of MAP of fish was published [15] however, there were only few references to research beyond the 90’s. A more current review of MAP for meat products was published [10], and includes considerations for commercial applications and an outlook on future needs and industry improvements desired for MAP of meat. The present review, as a result, only provides a brief overview of MAP principles with focus given to research on the use of modified atmosphere systems to fish and fishery products since 2000.

In 1990, Stammen defined MAP as a system where the air within a package is replaced by a mixture of different gases, where the proportion of gases in the mixture is fixed at the time of introduction and prior to final sealing of the package [14]. Once the package is sealed, however, there is no further control. For controlled atmosphere packaging (CAP), the composition of gases is continuously monitored and controlled throughout storage. Therefore, CAP systems maintain a stable gas composition as well as other environmental conditions within the package such as temperature and humidity [10].

For fish and fishery products, most gas mixtures do not include oxygen because high oxygen systems, in most cases, provide limited benefit to shelf life extension of fishery products [16,17,18,19]. This may be explained by the high rate of perishability of seafood, which results from psychrophilic spoilage bacteria growth that is combined with muscle degradation by endogenous enzymes and oxidative deterioration of the lipids, often rich in highly unsaturated fatty acids [20]. High oxygen concentrations, in combination with CO_2_, is used to preserve red color of haem pigments, and according to Davis, the main reason to include oxygen in the gas mixture of MAP fish and fishery products seems to be driven by a desire not to aggravate the potential risk of botulism from products in sealed packs [20]. In the Torrieri [18] study on the effect of combined use of MAP with an anti-oxidant based active packaging, containing α-tocopherol, on shelf life of bluefin tuna fillets stored at 3 °C for 18 days it was demonstrated that myoglobin oxidation was not reduced likely due to slow diffusion kinetics of the antioxidant from the film to the product surface. The authors also observed that oxidation was further aggravated by use of oxygen in one of the MA tested. On the other hand, Sivertsvik [21] reported significant improvement in the overall quality of MAP pre-rigor farmed Atlantic cod fillets by the use of high oxygen content in a gas mixture of CO_2_ and O_2_.

Fish and shellfish are highly perishable, and it is not possible to control many of the intrinsic and extrinsic characteristics of the products, and their respective harvest methods, that lead to reduction in quality and shelf life. For capture fisheries, the distance between harvest grounds and processing facilities and onboard storage temperature play major roles on product quality and deterioration. Low storage temperature significantly reduces the deterioration of product quality, however, time is a limiting variable because the spoilage of fresh fish is usually microbial [5]. As expected, at-sea processing [22] as well as prompt chilling followed by evisceration [23] tend to reduce quality deterioration of capture fishery products. Despite that, the high water activity of fish and shellfish combined with a neutral meat pH and the presence of autolytic enzymes further contribute to a high rate of perishability [5]. For aquaculture products, more control exists on the elapsed time from harvest to processing but other pre-harvest variables also influence product quality such as crowding stress [24] and disease. For these reasons, it is not surprising that some studies report limited benefit to the quality and shelf life of fish and fishery products by the use of gases mixtures in MAP [25,26,27,28], while many others record significant extension in product shelf life [29,30,31,32,33,34,35].

## 2. Modified Atmosphere Systems

The primary gases used in MAP of seafood products are CO_2_, N_2_ and O_2_. The deleterious effects of oxygen on the quality of stored fish and fishery products have been well described in the literature. The reduction of oxygen slows down lipid oxidation and the development of rancidity. For seafood products, the simple flushing with nitrogen is used as an alternative to vacuum packaging to replace O_2_ in packages to delay oxidative rancidity and inhibit growth of aerobic microorganisms. However, for the most part this gas is used in gas mixtures of MAP as a filler (e.g., CO_2_:N_2_) because of its low solubility in water and fat, which prevents packaging collapsing [5]. CO_2_ has been long known for its fungistatic and bacteriostatic properties in food systems [36] and is, therefore, the most important gas used in modified atmosphere systems of seafood [5].

Vacuum packaging was the first commercially developed MAP and consists of packaging a product in a film of low oxygen permeability and sealing it after evacuation of air, which under good vacuum conditions should reduce oxygen concentrations to levels below 1% [37]. This review intends to discuss MA systems in which gas packaging is used. For modified atmosphere systems, the modified atmosphere can be achieved by mechanical replacement of air with a single gas or a gas mixture, or by generating the atmosphere inside the package either passively or actively [37]. Passive generation of atmosphere inside a package is applicable to respiring foods, while active atmosphere generation may be accomplished by the use of O_2_ scavengers, CO_2_ absorbents or emitters, ethanol emitters and ethylene absorbers among others [37].

The mechanical replacement of air may be achieved either by gas flushing or compensating vacuum. Gas flushing is generally achieved using form-fill-seal equipment that injects a continuous stream of gas, or mixture of gases, into the package [37]. When the air has been replaced by the gas via a dilution process, the package is sealed. Residual oxygen levels in gas-flushed packs vary according to equipment used, type of packaging and product type. Blakistone points out that the typical residual oxygen levels in gas-flushed packs are in the range of 2% to 5% [37]. Hence, this technique alone is not suitable for packing of foods that show high rate of deterioration by the action of oxygen. Another technique for mechanical replacement of air is compensated vacuum, which consists of a two-stage processes where the air is first removed by the action of vacuum and then the modified gas atmosphere is introduced prior to sealing the packaging [37]. The packaging can be pre-formed or thermal formed, and the efficiency of the process is much superior to that achieved by gas flushing with regards to residual oxygen levels [37].

Bulk storage for transport of foods in MAP and CAP is widely used by the food industry, but only a few studies describe bulk storage of seafood products under modified atmospheric conditions [38]. Fish and fishery products can be placed in a master pack containing multiple units of individually packed product, which are overwrapped with either PVC film or other type of gas permeable film. The master pack consists of a large partial barrier film or pouch bag that is flushed with the desired gas mixture, which generally includes high levels of CO_2_ [1]. Prior to retail display the consumer package is removed from the master package. A study on bulk storage of fish under modified and controlled atmospheric conditions was conducted by Ruiz-Capillas and Moral in which the authors compared the residual effect of CO_2_ on the shelf life of whole gutted hake kept in boxes of ice for 12 days onboard of a fishing vessel under atmosphere composed of different mixtures of CO_2_:O_2_:N_2_ [38]. When the vessel reached port, the containers were opened and the products were stored on ice until spoiled. The authors concluded that the controlled atmosphere mode was significantly more effective in keeping product quality and extending product shelf life than the modified atmosphere [38]. The lots of whole gutted hake that scored best in sensory analysis were those kept under controlled atmosphere in the gas mixtures composed of either 60% or 40% of CO_2_ [38]. Overall, the vast majority of the studies for fish and fishery products apply MA to retail-type package. Shelf life of MAP fillets from a wide variety of fish species have been determined and are summarized in Table 1 for research published since 2000. Consistently a common variable for a majority of these studies is that product is packaged in retail-type trays such as for Atlantic cod [6,7], Atlantic horse mackerel [3,39], Atlantic salmon [24,40,41], Bluefin tuna [18], dolphin fish [42], sea bass [33], and seer fish [43] among others.

## 3. Solubility of CO_2_ in Fish and Fishery Products

CO_2_ is by and large the most studied gas in MAP of fish and fishery products. Its inhibitory effect on the growth of microorganism is concentration dependent, and the higher the concentration of CO_2_ in the system the higher the inhibitory effect observed [5]. The inhibitory effect of CO_2_ is related to its high solubility in both the water-phase and lipids of muscle foods. The lower the temperature, the higher the solubility. Knoche determined the solubility of CO_2_ in 1 kg of water (1 atm) at a temperature of 0 °C to be 3.38 g, while it is virtually 50% less soluble than that (1.73 g/kg) in 20 °C water (1 atm) [44]. The reaction scheme of dissolved CO_2_ in water is equimolar and generates a bicarbonate ion and one proton or a bicarbonate ion and two protons, with the formation of the dissociated ions being pH dependent. For pH values less than 8, as for most seafood, the concentration of carbonate ions is negligible [5].

The solubility and absorption rates of CO_2_ into non-respiring foods such as raw fish [45] and cooked meats [46] has been determined using a validated experimental apparatus that applied a manometric method [47]. The method was developed using an experimental apparatus for determination of the solubility and the absorption rate of CO_2_ into foodstuffs indirectly by continuous logging of pressure changes in a closed chamber [47]. The authors tested several species of fish and, in particular for Norwegian cod and salmon samples, they investigated the effect of a variable ratio of weight of product to gas volume that ranged from 1.5 to 5, with gas mixes of 25%, 50%, 75% and 100% CO_2_, at discrete temperatures in the range of −2 to 4 °C. The authors concluded that the solubility of CO_2_ in raw fillets of salmon, cod, anglerfish, wolf fish and tuna, can be predicted using the solubility of CO_2_ in water corrected for the water and lipid content of the sample [45]. Among other important observations, Sivertsvik noted the average time to reach 50% of the equilibrium solubility for all their experiments was 4.6 ± 0.4 h, with equilibrium not reached until about 3 days (68.9 ± 6.7 h) [45]. This has profound consequences to MAP systems because changes in the composition of the headspace may occur over a relatively long period of time after packaging, depending on the ratio of gases used, with fluctuations in product temperature further influencing the state of equilibrium of CO_2_ in the product versus in the gas phase. A direct application of their findings to MAP of fish is that a fillet can be exposed to 100% CO_2_ in MAP for about 4.6 h or to 50% CO_2_ for about 3 days to obtain the same amount of dissolved CO_2_ in the product, hence a similar effect on shelf life extension [45].

A subsequent study published by Rotabakk [48] proposed a volumetric method to determine CO_2_ solubility and absorption rates in foods packaged in flexible and semi-rigid package. The headspace volumes of the packages were determined by submerging the package under water and measure the resultant force by a texture analyzer. Measurement of the headspace volume was performed by first submerging the equipment to determine its volume. The package was then submerged and held upside down until the system stabilized and buoyancy force measurements were taken at set intervals and correlated to headspace volume changes [48]. The determined headspace volume changes were well correlated to the determined solubility and absorption rate of CO_2_ in packages determined by monitoring pressure changes that were assessed using a manometer [48]. The effectiveness of MAP in shelf life extension of foods is dependent on the amount of CO_2_ that can dissolve in the product, which in turn is dependent on the partial pressure of CO_2_ within the package and the degree of filling (DF) described as volume of product to volume of packaging. Given that many studies on shelf life extension of seafood do not state either the DF or the amount of dissolved CO_2_ in the product, direct comparison of results becomes difficult [48]. The method proposed by Rotabakk [48] is non-destructive and provides a simple solution to monitor the changes in the headspace volume inside the package over time.

Fernandez [40] estimated the solubility of CO_2_ at equilibrium, using an array of gas compositions (CO_2_:N_2_) for superchilled (−1.5 °C) MAP Atlantic salmon fillets stored at 2 °C. The authors applied either a ratio of gas to product (g/p) of 1.2 or 2.5. For products packaged with a g/p of 1.2 the estimated solubility of CO_2_ for the best performing gas mixture (75:25) was 797 ppm. For this packaging and storage condition, based on microbial (TPC < 10^6^ cfu/g) assessment, the shelf life of salmon fillets was 18–19 days while product maintained acceptable sensory parameters for 27 days of storage [40]. For products packaged with a g/p of 2.5 the estimated solubility of CO_2_ for the best performing gas mixture (90:10) was 2065 ppm. For this packaging and storage condition, based on microbial (TPC < 10^6^ cfu/g) assessment, the shelf life of salmon fillets was 22–23 days while product maintained acceptable sensory parameters for more than 28 days of storage [40]. This study demonstrates the importance of determining CO_2_ concentration in MAP products for optimization of product shelf life.

## 4. Preservation Effect of CO_2_ in Fish and Fishery Products

Literature findings clearly show individual bacteria vary in sensitivity to CO_2_ however, as stated by McMillin gram-negative bacteria are generally more sensitive to CO_2_ than gram-positive bacteria because a large number of gram-positive bacteria are either facultative or strict anaerobes [10]. Despite research efforts on the identification of specific spoilage organisms (SSOs) for fish and fishery products stored under modified atmosphere conditions, the breadth of knowledge of SSOs of fishery products from different aquatic environments is still limited, especially when different packaging conditions are taken into consideration [5]. Some examples of SSOs identified MAP spoiled fish are *Photobacterium phosphoreum* for MAP cod fillets [49], *Serratia liquefaciens* (*Serratia protamaculans*) for vacuum packaged cold-smoked salmon [50], *Carnobacterium*, *Serratia*, *Shewanella* and *Yersinia* for MAP horse mackerel fillets [4,41], *Carnobacterium*, *Lactococcus* and *Vagococcus* for MAP Australian salmon [51], and *Hafnia alvei* in MAP Atlantic salmon [52]. Given the diversity of fishery products and associated SSOs, modelling the effects of MAP variables (temperature, gas mixtures, product to gas ratio, concentration of CO_2_ in product, and degree of filling among others) on microbial growth is highly desirable for advancements in the field. Alfaro, Powell, and Chaix provide comprehensive information on existing predictive models relating MAP conditions, type of fish/fishery product and growth rate of associated SSOs [4,9,53].

The preservative effect of CO_2_ on the growth inhibition of microorganisms in foods is a complex process that cannot be solely explained by either the change in pH derived from the solubility of the gas in the product, or the displacement of some or all the oxygen available for bacterial metabolism. In their review of MAP in fish, Stammen [14] fully describe research findings relevant to the mechanisms and effects of CO_2_ in microbial inhibition such as the synergism of CO_2_ concentration and temperature, the lengthening of the microbial growth lag phase under controlled environmental conditions, and the potential inhibitory effect on enzymatic activity. A decade later, Siverstsvik [5] summarized four fundamental mechanisms of CO_2_ on microorganisms proposed by various researchers, (i) penetration of CO_2_ in the membrane of bacteria that results in intracellular pH changes; (ii) alteration in cell membrane function that results in deleterious effect on nutrient uptake and absorption; (iii) direct inhibition or retardation of enzymatic activity, and iv) direct changes in the physical and chemical properties of proteins. In a recent publication by Chaix [53], the authors stated that in spite of the many hypotheses proposed to explain the inhibitory effect of CO_2_ on bacterial growth, present information is still unsatisfactory to allow for a complete understanding of the underlying mechanisms albeit highly important to future advances in MAP and CAP of foods.

## 5. Quality of Raw Material for Modified Atmosphere Applications

Shelf life extension of fish and fishery products by the use of modified atmosphere systems can only be effectively achieved when the highest quality raw material is utilized [5]. This concept, albeit simple, is at times difficult to follow given that fish quality is affected by a large number of factors beyond time and temperature such as harvest season, catch method, slaughter method, species-specific biochemical characteristics, environmental conditions, spawning and overall health of the animal. Application of MAP or CAP to fish does not improve poor quality product nor does it give back lost shelf life. On the contrary, chemically and/or enzymatically degraded product and the presence of high levels of spoilage bacteria will lead to either minimal shelf life extension or to no extension at all. Compounded to this issue is the increased risk of product contamination with pathogens that may result from temperature abuse and/or poor handling and sanitation practices of raw material, which may pose serious risk to consumers.

Beyond compliance to all seafood safety requirements and HACCP, the freshness of raw material is of primary importance to assure only high quality seafood is utilized for MAP or CAP applications. As previously stated by Huss, fish quality refers to the aesthetic appearance and freshness or degree of spoilage which the product has undergone [54]. It may also involve safety aspects such as being free from harmful bacteria, parasites or chemicals. Quality of seafood is a very complex concept [55], for instance Botta cites 15 different definitions of quality [56]. Although the concept of quality is frequently described using terms related to nutritional, microbiological, biochemical and physiochemical characteristics, none of these terms serve as adequate indices of sensory perception and consumer acceptability [55]. Nevertheless, many of the quality issues observed in consumer ready fish and fishery products derive from the initial quality of the fresh raw material, which starts declining immediately post-mortem [57]. Some important fish handling and quality guidelines that should be followed from harvest to processing of fresh fish to realize the maximum benefit of MAP or CAP are:

- Stressful conditions during harvest and slaughter should be avoided to maximize eating quality of fish [24,58];
- Fish should be bled promptly, and eviscerated as soon as possible [23];
- Fish should be promptly chilled and maintained at a temperature approaching that of melting ice from time of slaughter to processing [59];
- Appearance of the eyes, gills and skin should be evaluated to conform with high quality attributes as described in methods for sensory evaluation of fish and fishery products such as the Torry Fish Quality Assessment System [60,61], the Quality Index Method [62] among others [54,63]. Other important characteristics are:
  - Fish should have firm to normal texture (not soft) and odor notes typical of fresh fish (ocean-breeze, briny, cantaloupe, melon and cucumber notes) should be present;
  - For fillets, flesh color should be uniform at the typical color range for the species;
  - Fillets should have little to no gaping.
- For headed and gutted fish, it is important the kidney is thoroughly removed and that the belly cavity is free of any blood, viscera and debris, and without noticeable structural damage to belly cavity membrane;
- For descaled fish, the descaling process should be conducted using a method that does not cause excessive flesh softening and subsequent muscle gaping;
- The removal of pin bones from fillets, when applicable, should be carried out using either a manual or mechanical method that does not cause excessive muscle tearing, which could result in gaping;
- The initial microbiological load in fresh fish from capture fisheries varies widely with values that may exceed 5 Log_10_ cfu/g, depending on onboard sanitation and handling practices [64]. The importance of a low bacterial load, preferably at the range of 2 to 3 Log_10_ cfu/g, has been identified as key factor for maximizing the shelf life extension of seafood packaged in MA systems [41,65,66,67].
- Processing fresh high quality product under hygienically good conditions [65,66] must be underscored because finished products with high microbial load (e.g., 5–6 Log_10_ cfu/g) are not likely to benefit from MAP. As stated by Farber over two decades ago, there is no enhancement of product quality because MAP simply arrests the natural deterioration process [1].
- It may be desirable to subject the finished product to a final sanitation step prior to MAP. Sanitation of finished product may consist, among others, of application of aqueous ozone, electrolyzed water, aqueous solution of peroxyacetic acid or sodium hypochlorite, and the sanitizer choice will depend on local regulatory requirements and the achievable reduction of surface bacteria by the selected treatment and mode of application.

The application of modified atmosphere to pre- versus post-rigor fish is a topic of interest for packaging of aquaculture fish because it is possible to manage many of the variables influencing onset, duration and resolution of rigor while this is often not the case for fish captured from the wild. Withdrawing of feed prior to slaughter, slaughter method, elapsed time between slaughter and processing, and holding temperatures post-slaughter are important variables influencing rigor mortis process that can be managed for the processing of aquaculture fish pre-rigor [41,66,68]. According to Hansen, an increased interest exists in pre-rigor filleting of farmed fish because such early processing, accomplished under hygienically good conditions, allows super-fresh products to enter the market [41]. This improves the logistics at the processing plant and eliminates costs associated with transportation of head and bones [41]. The authors also noted pre-rigor salmon fillets are thicker and firmer, and have a more intense coloration as compared to their post-rigor counterparts [41]. Sivertsvik determined the optimal initial gas composition to maintain quality of modified atmosphere packaged pre-rigor filleted farmed Atlantic cod, stored up to 14 days at 0 °C, to 37:63 (*v*/*v*) CO_2_:O_2_ [21]. The author concluded that the optimal gas mixture yielded the lowest formation of exudate, TMA and total volatile bases, while having the highest inhibitory effect on microbiological growth; and at the same time maintained a high odor score and TMAO content in packaged farmed pre-rigor cod fillets [21]. Irrespective of the advantages highlighted above on the application of MA systems to pre-rigor fish, for products from capture fisheries there is limited possibility of applying modified atmosphere technology to pre-rigor fish unless fishing grounds are at close proximity to processing plants, or fish is processed and packaged at sea onboard of catcher-processor vessels. In general, fishery products processed at sea are not packaged in consumer-ready packaging because space limitations exist for storage of materials and finished products, and bulk packaging of frozen products offer significant advantage for preservation of quality.

## 6. Effect of Processing and Storage Temperatures

Storage temperature of seafood has a dramatic effect on product shelf life despite packaging method. For studies applying MA systems to fish and shellfish, a wide range of storage temperatures are reported and include those much beyond the optimal range for seafood storage even for products aerobically packaged. This is evident from the wide range of product storage temperatures provided in Table 1, which lists selected literature findings on the application of MA systems to fish and fishery products through the past 15 years. One obvious reason researchers have for choosing high storage temperatures of MAP products (>3 °C) is the assessment of consumer safety and risks. A second reason for this choice is based on the fact that many retail establishments are simply not capable of maintaining seafood at the adequate low temperature range. Finally, even when the product cold-chain is not broken from harvest to retail, there is a high risk this could happen during refrigerated storage at home. These points underscore the need for consumer-ready MAP products that either allow oxygen exchange by the use of permeable films, or that contain sufficient oxygen in the gas mixture to prevent the potential risk of botulism.

It is well established that holding seafood at the superchilled temperature range, which is defined as the range of temperature below 0 °C and slightly above the temperature in which freezing is discernable [69], leads to longer shelf life extension than that achieved for product held under optimal refrigerated temperatures [65]. As stated by Davis, fish spoil more than twice as fast at 5 °C than at 0 °C, and four times as fast at 10 °C [20]. Likewise, a reduction in the rate of spoilage is observed when product is held at slightly above freezing temperature, within the range of −1.0 to −3.0 °C, when compared to being held at temperature of melting ice as it was demonstrated for Atlantic cod by Duun and Rustad [70]. As previously stated, a large number of variables play a role on the level of shelf life extension that can be achieved by the application of MAP and CAP to fish and fishery products. A comparison of literature results is, therefore, a complex task given the wide range selected for these critical variables by the authors, and in some instances the lack of information regarding ratio of gas to product, amount of dissolved CO_2_ in the product, and variability of gas composition in the package headspace during storage. A quick glance through Table 1 allows one to conclude that a majority of the studies reporting product storage temperature above 0 °C are confined to the determination of product shelf life for up to 3 weeks, with few studies having a duration of 4 weeks or beyond. For products stored at temperatures above superchilling, the shelf life extension provided by MAP may range from a few days to a week or more compared with air storage, depending on species and temperature [65]. On the other hand, the use of superchilled storage temperatures has opened a new avenue to extend the shelf life of MAP products to four weeks and beyond.

Used alone, the storage of fish and fishery products at the superchilled temperature range is an established technique to extend shelf life of air-packed products [126]. According to Duun and Rustad, the storage time of vacuum packed salmon fillets can be doubled by superchilled storage at −1.4 °C compared to ice chilled storage [127]. Kaale and Eikevik noted that the quality and freshness of the final superchilled food are highly influenced by superchilling rates and degree of superchilling [69]. The degree of superchilling being defined as the amount of free water frozen (5% to 30%) inside the food product [128]. Other important variables affecting final quality of superchilled products are storage temperature and time, type of product, and packaging selection [69]. Superchilling of foods is generally regarded as effective and practical, provided that the product temperature does not fall below the point in which freezing is discernible [69], for most foods the initial freezing point falls between −0.5 °C and −2.8 °C.

For pre-rigor Atlantic salmon fillets that were superchilled in a tunnel operated at −25 °C for 45 min and subsequently stored at −1.5 °C, the formation of intra- and extracellular ice crystals accelerated lysosome breakages followed by release of cathepsin B and L [129]. In this case, superchilling resulted in higher liquid leakage and increased myofiber breakages in the fillets. Texture values measured instrumentally, however, were not affected by superchilling one week after treatment [129]. In a later study, Kaale and Eikevik demonstrated that both, shell freezing and the characteristics of ice crystals (location and distribution) strongly influence drip loss and overall eating quality of superchilled salmon fillets [128]. Shell freezing consists of subjecting product to sufficiently low temperature to allow initial surface freezing before subjecting the final product to a storage temperature in the superchilled range [128]. The authors recorded a significant difference in the drip loss between shell frozen samples (1.6%) and non-shell frozen samples (1.4%). Furthermore, they determined that for non-shell frozen samples ice crystals were exclusively of the extracellular kind, while shell frozen samples showed both intracellular and extracellular ice crystals with a uniform distribution [128]. Overall, the values between 1% and 2% of drip loss cannot be regarded as high and cannot be considered as a major problem in superchilled salmon. There was also no significant difference between non-shell frozen and chilled samples, despite that, the authors stressed the importance of process optimization to minimize damage to muscle structure due to ice crystal formation [128].

For MAP fish and fishery products stored in the superchilled temperature range, optimization of process variables is also a key step to preserve muscle structure integrity and minimize muscle softening and drip loss, which leads to deterioration of product appearance and facilitates surface microbial growth [127]. Sivertsvik demonstrated that combining superchilling storage (–2 °C) with MAP (CO_2_:N_2_ 60:40) of Atlantic salmon fillets extended product shelf life by four fold when compared to air packed product kept under refrigerated temperature (4 °C) [65]. In their study the superchilled MAP fillets maintained good quality with negligible microbial growth (<1000 cfu/g) for more than 24 days, the air packed superchilled fillets had 21 days of shelf life, while air-stored and MAP fillets kept at chilled conditions (4 °C) spoiled after 7 and 10 days, respectively. Fernández [40] achieved slightly shorter shelf life results than those reported by Siverstvik [65], for Atlantic salmon fillets superchilled (−1.5 °C) in MAP with a CO_2_ concentration of 90% and gas to product ratio of 2.5. The shorter shelf life is likely due to a higher post-superchilling storage temperature of 2 °C for the shelf life study [40]. Hansen determined the shelf life for pre-rigor fillets of Atlantic salmon superchilled prior to packaging in modified atmosphere (CO_2_:N_2_ 60:40) with a CO_2_ emitter that were subsequently stored at 0.1 °C for 28 days [41]. The chilled fillets stored in MAP had significantly lower bacterial growth compared to fillets stored in air, and MAP superchilled fillets had lower bacterial counts compared to the corresponding chilled fillets [41]. Samples superchilled prior to refrigerated storage in air had similar bacterial growth to the ordinarily chilled samples, indicating that superchilling treatment followed by chilled storage temperature does not extend shelf life. The range of shelf life for MAP Atlantic salmon fillets observed by Fernández, Hansen, and Sivertsvik depict the strong influence of storage temperature on product shelf life with small increases in temperature of 1 °C leading to a reduction of 2 or more days in shelf life [40,41,65].

Wang investigated the effect of combined application of MAP (CO_2_:N_2_:O_2_ 50:45:5) and superchilled (−0.9 °C) storage on the shelf life of fresh cod loins [6]. Superchilled storage alone compared with traditional chilled storage, in polystyrene boxes, increased the total shelf life of cod loins from 9 to 16–17 days (from catch date). Chilled (1.5 °C) MAP increased the shelf life from 9 to 14 days, and when MAP and superchilled storage were combined the shelf life (from catch date) was further extended to at least 21 days [6]. The authors noted that MAP combined with superchilled storage preserved the characteristic fresh and sweet taste of cod loins for a longer period of time, which enhances eating quality of fresh cod fillets for consumers in distant markets. However, some different textural properties were recorded, and after only 7 days of storage the superchilled MA packed cod loins had a meatier texture compared to other sample groups [6]. In a follow up study, Lauzon evaluated the effect of brining (~2.5% NaCl), MAP (CO_2_:N_2_:O_2_ 50:45:5), and storage temperature (0, −2, and −3.6 °C) on the quality changes of fresh cod loins, packed 4 to 5 days post-catch, over a period of 4 weeks [7]. The shelf life of air-packed loins was about 11 and 14–15 days at storage temperatures of 0 and −2 °C, respectively. The shelf life of MAP cod loins stored superchilled (−2 °C) increased to a total of 21 days from catch date. Interestingly, for brined loins the shelf life of air-packed and MAP product stored superchilled was 12 to 15 days and 13 days, respectively. In summary, the addition of salt to cod loins led to a slight reduction of shelf life when product was stored in MAP; however, for cod loins without the brine treatment the shelf life increase was substantial. A follow up study by Gudjónsdóttir [77] confirmed Lauzon [7] findings.

Zeng showed that the total viable counts of bacteria increased rapidly in shrimp stored in flake ice and in brine mixed with flake ice, followed by those in liquid ice at +1.5 °C, while slow bacterial growth and low counts were observed in shrimp stored in superchilled liquid ice (−1.5 °C) [130]. An noteworthy study published by Bono recorded the effects of modified atmosphere packaging (MAP) using two different gas compositions (100% N_2_ or CO_2_:N_2_ 50:50) on the chemical properties and melanosis of deep-water rose shrimp over a one-year frozen storage period [85]. The authors recorded inhibited lipid oxidation and low melanosis scores for frozen samples packed in 100% N_2_ up to six months of storage.

## 7. Microbiological Considerations

A major challenge for the seafood industry is the control of bacteria, pathogenic or spoilage specific, in fresh products. Farber in his review of microbiology of MAP points out that one of the key safety issues of MAP is not just psychrotrophic, non-proteolytic clostridia, but the emergence of other psychrotrophic pathogens such as *Listeria monocytogenes*, *Aeromonas hydrophila*, and *Yersinia enterocolitica* [1]. Farber points out that the inhibition of spoilage microorganisms and extension of shelf-life by MAP may allow the psychrotrophic pathogens sufficient opportunity for growth [1]. For fresh meat systems, however, it is noted that under CO_2_, the system switches from initial gram-negative aerobic spoilage to a “predominantly gram-positive facultative anaerobic microflora dominated by *Lactobacillus* spp.” This switch, however, was viewed as positive because the byproducts of metabolism of lactobacilli are slowly produced and less offensive compared to the pseudomonads.

The research focused on pathogens since 2000 has evaluated MAP for its effect on *Listeria monocytogenes* [73,106,107,121], *Staphylococcus aureus* [28,99,107], *Salmonella* spp. [28,73,100,107], sulfite-reducing *Clostridium* [28], nonproteolytic *Clostridium botulinum* [106], and *Clostridium botulinum* toxin production [80,99]. The storage of product in MAP was noted to not increase the risk of pathogen growth for *Staphylococcus aureus* above that of air packaged products [99]. *Listeria monocytogenes* was reported as controlled in RTE pink shrimp when temperature was kept below 2 °C. It was also observed that frozen storage prior to chilled storage was effective in limiting growth of *L. monocytogenes*, but it did not inactivate it. It was also demonstrated that *L. monocytogenes* was the greatest risk for brined and drained shrimp, followed by *S. aureus*, *Salmonella* spp., and *Vibrio parahaemolyticus* in descending order [107]. A survey of 90 carp portions [73] stored at 4 °C reported no *L. monocytogenes* or *Salmonella* spp. up to 10 days at which time all products were determined to be in the spoiled state and past their shelf-life. Yilmaz, observed MAP conditions delayed *L. monocytogenes* growth [121]. Soccol noted that *S. aureus* was not significantly affected by the atmosphere treatments they studied [28]. They also reported that *S. aureus* decreased over time for all treatments, except vacuum. They also did not detect *Salmonella* spp. nor sulfite-reducing *Clostridium* in any of the treatments. Zhou, found a synergistic effect in *Salmonella* spp. inhibition when MAP was combined with an antimicrobial, vapor phase thymol [100]. *Clostridium botulinum* toxin production was prevented in fresh fish fillets by keeping temperatures below 2 °C and having 30% O_2_ in the MAP mixture [99]. Toxin production was also prevented at 4 °C using 3000 cm^3^·m^−2^·24 h^−1^ oxygen transmission rate (OTR) film [80]. In a CO_2_ only atmosphere, however, toxin was detected after 25 days at 4 °C and 9 days at 10 °C. Toxin production preceded spoilage (>35 days) at 4 °C and occurred at the same time as spoilage when the temperature of storage was 10 °C [80].

Fresh fish spoilage is typically attributed to psychrotolerant bacteria, such as *Pseudomonas* spp. [22,131]. As early as 1933, Coyne noted that *Pseudomonas* spp. can be controlled by as little as 5% CO_2_ [131]. The reduction of total viable counts as a result of including CO_2_ in the gas mix has been further confirmed in many of the studies in this review encompassing the following species/products listed in Table 1: bonito [72], cod [6,21,22,30,76], crayfish [79], halibut [81], lingcod [82], mussels [87], mullet [85], prawn [88], salmon [25], sardines [32], scallops [93], sea bass [26,96,112], seabream [19,95], shrimp [31,100,103,113,114,116], squid [118], tilapia [28], and trout [119,120,123].

*Photobacterium phosphoreum* is described as CO_2_ resistant and is the main spoilage organism associated with MAP packaged cod and other high TMAO species [5,132,133]. Fletcher however, reported low levels of this organism in MAP packaged king salmon and suggested it might not be a spoilage organism of concern for New Zealand [25]. Similarly, Milne and Powell, examined the dynamics of microbial growth in fresh chilled Atlantic salmon (*Salmo salar*) farmed in Australia [67]. Atlantic salmon, less than 24 h from harvest, were used to produce skinless fillet portions packed in pouches containing 96% CO_2_ at gas: product ratios of greater than 5:1 (*v*/*w*) and stored for 38 days at less than 1 °C. As with the study from New Zealand, the authors reported that *Photobacterium* spp., including the specific spoilage organism *Photobacterium phosphoreum*, were not identified during their study [67].

In an interesting study on the effects of storage prior to MAP packaging and retail display of cod, Bøknæs demonstrated that −30 °C storage prior to MAP packaging actually helped *Photobacterium phosphoreum* survive better than −20 °C storage [22]. While −20 °C stored fish had <1 log cfu/g for up to 17 days in MAP, −30 °C stored fish had between 10^4^ and 10^6^ cfu/g by day 17. They also demonstrated that storage at 0 °C for 8 days prior to freezing at −30 °C would result in significantly high TMA values in cod. Surprisingly, TMA values in similarly chilled stored cod (i.e., 8 days) that were subsequently stored at −20 °C prior to MAP packaging were not any different than products stored at 0 °C for 1 day prior to frozen storage at either −20 or −30 °C. Fletcher et al., 2004, for king salmon also reported there was not an effect of level of CO_2_ in MAP or ratio of fish to CO_2_ on production of TVB-N. They also reported that TVB-N increased over time regardless of fish: CO_2_ ratio. They did not, however, compare these increases over time with an aerobic packaged product. They only looked at the TVB-N in aerobic packed fish on day 0. Özogul, however, did compare air, vacuum and MAP sardines and reported TVB-N correlated strongly with total viable counts, regardless of storage conditions [32]. In MAP cooked pink shrimp, formation of both TVB-N and TMA were reported to be negligible even at spoilage (determine from sensory decomposition evaluations) [106].

Fletcher reported that at 90 days of chilled storage, vacuum packed king salmon were dominated by *Serratia liquefaciens* which decreased as CO_2_ increased, with *Lactobacilli* spp. predominating at high levels of CO_2_ [25]. They noted that S. liquefaciens produced worse spoilage odors than *Lactobacilli* spp and also reported the virtual absence of sulfide-producing organisms at day 90. Mejlholm reported that lactic acid bacteria dominated the spoilage of microflora of MAP pink shrimp [106]. An important take away from this research is how MAP can be used to perhaps selectively alter the spoilage microflora in a way that minimizes the intensity of the degradative actions of the microbiota on fish quality.

## 8. Additives and Shelf Life Extension

Several researchers report on the use of natural or synthetic additives to enhance quality and shelf life of MAP products. In some cases, researchers have gone as far as to conclude that the combination of MAP with certain antimicrobials or antioxidants may be synergistic [98,100,110,116,117]. Additives investigated with MAP since 2000 include acetic acid, benzoic acid, bi-sulfite, cinnamic acid, citric acid, chitosan, electrolyzed water, 4-hexylresorcinol, krill oil, lactic acid, nisin, oregano essential oil, ozone, quercetin, sodium acetate, sorbic acid, thymol, and extracts from halophyte plants (poylphenols including flavonoids, caffeic acid esters, and coumarins), grapefruit seed, and green tea extract and UV-C radiation [18,28,30,42,82,85,95,96,98,99,100,107,110,111,112,116,117,122]. In almost all cases, beneficial effects are observed when MAP is combined with another known additive that typically is added to either act as an antimicrobial or antioxidant. The exception was notably the application of UV-C radiation in yellowtail tuna [34]. Researchers did not see an extension in shelf life and, as expected, oxidation was concomitantly increased. A negative effect on odor and volatile flavor was also reported for the use of oregano essential oil and electrolyzed water [117,122]. While both treatments did decrease bacteria and increase shelf life, the former was found to be unsuitable at 0.4% due to odor, the latter was found to create an odor issue (chlorine) only in the thawed raw product. Researchers said the chlorine odor in thawed product did not create a noticeable effect in the cooked product.

## 9. Physicochemical and Organoleptic Considerations

Many important quality changes observed in MAP seafood are directly related to microbial deterioration and may result in off-odors, off-flavors, muscle softening, discoloration, and an increase in muscle exudate during storage. Quality changes may also derive from the incorporation of CO_2_ to the aqueous phase of the food, which causes a reduction in muscle pH and in protein water retention ability, causing an increase in drip loss during storage. Texture degradation of MAP products may also occur due to endogenous enzymatic activity, which may increase by the drop in muscle pH caused by CO_2_.

Nucleotide degradation is the first biochemical change observed in post-mortem muscle, which is tightly connected to rigor mortis process. Despite its importance for quality assessment of fresh seafood, quantifying ATP decomposition or measuring K value is of little use for quality assessment of MAP products, even for fish packaged pre-rigor. This is mainly due to the fact that research has shown that CO_2_ atmosphere does not alter the K-values compared with those determined for product aerobically held [5,94,125].

Total Volatile Basic Nitrogen (TVB-N) is a chemical indicator often used to determine freshness of fish and fishery products held on ice. Determination of TVB-N values and Trimethylamine (TMA) content are widely used in shelf life studies of MAP seafood [6,25,32,38,88,99,123]. Some authors determined a correlation between quality deterioration of MAP seafood and increased TVB-N or TMA values, but often noted the levels were below limit for consumption at the end of the product shelf life [32,123]. Others observed significantly lower production of volatile bases for products in MAP compared to their air-packed counterparts [6,38,99]. Bøknæs determined that spoiled MAP Atlantic cod is characterized by high levels of TMA because its prevalent spoilage organism is *Photobacterium phosphoreum*, which can reduce trimethylamine oxide (TMAO) to TMA and cause development of fishy odor [75]. Similarly, Alfaro determined TMA to be a good indicator for spoilage of MAP of Atlantic horse mackerel [3]. Emborg noted the formation of biogenic amines was limited in fresh MAP salmon [91]. In a later study, Yew also recorded inconsistencies in the formation of biogenic amines in MAP Indian mackerel as compared to air packed control samples [84]. The usefulness of TVB-N values, and levels of TMA or specific biogenic amines, including histamine, as spoilage indicators for determination of shelf life of MAP fish and fishery products depends on the specific spoilage organisms that dominate the microbial flora during product degradation.

For some MAP products the end of shelf life is determined by sensory rejection and before microbial levels indicate spoilage (e.g., TVC ~ 10^5^–10^6^ cfu/g). In these cases, non-microbial degradation drives end of shelf life and sensorial changes at point of rejection may be due to changes in odor, taste, texture, color, or a combination of these variables. For this reason, a majority of studies addressing shelf life of MAP of fish and fishery products include sensory assessment and Table 1 provides several examples of MAP products that reached end of shelf life by sensory rejection.

Changes in pH are often recorded in shelf life studies of MAP products and may correspond only to a slight decrease or to a much larger drop from pH 7.0 to 6.0 [3,8,41]. A subsequent increase in pH during product storage may correspond to microbial activity [6], but this variable alone is not a useful shelf life predictor because it may not always correlate well with bacterial growth [3]. Many studies indicate a direct correlation exists between CO_2_ concentration and liquid loss [41] because CO_2_ tends to decrease water holding capacity [5], but the magnitude of the liquid loss appears to depend on storage temperature and type of product [65]. Drip losses in the range of 2% to 6% are well within values reported in the literature for MAP of fish [6,7,40,41,65].

Oxidative rancidity may also be a limiting factor in the shelf life of MAP seafood, especially for fatty fishes [5]. The usefulness of thiobarbituric acid values (TBA) and thiobarbituric acid reactive substances (TBARS) as an indicator of shelf life depends on the type of product. For instance, Alfaro determined that TBA was not a useful indicator for shelf life determination of MAP of Atlantic horse mackerel, while Torrieri found TBARS to be a critical index for determination of shelf life of MAP of Bluefin tuna [3,18].

Texture is an important quality parameter for seafood but the changes in texture associated to storage under MAP are not fully understood. Masniyom investigated the degradation of proteins in seabass muscle during MAP storage [94]. The authors recorded a decrease in total sulphydryl content with a concomitant increase in surface hydrophobicity in samples stored under MAP, compared to those kept under air atmosphere. However, no marked autolytic degradation in samples kept under MAP was observed throughout the storage [94]. Hansen reported a decrease in firmness of MAP Atlantic salmon fillets packaged pre-rigor by measuring force required to penetrate through the fillet [41]. Interestingly, the author reported that MAP seemed to reduce the release of two important lysosomal enzymes responsible for tenderization of fish muscle, cathepsin B and L, after 7 days of storage for chilled and superchilled MAP salmon fillets [41]. Chen recorded a reduction of calpastatin, the endogenous calpain inhibitor, in MAP stored crayfish samples after 10 days [29]. This reduction, albeit suggestive of high m-calpain activity did not correspond to softening of crayfish, on the contrary, an increase in toughness was observed after 10 days of storage [29].

## 10. The Future of MA Systems

Advances in the application of MAP to shelf life extension of muscle foods is occurring at a fast pace. Research over the past 15 years has increasingly focused on a more sophisticated systems-based approach to MAP development and evaluation in fishery products. Considerable more research is needed to fully understand how MAP, temperature, and additives can be employed to selectively target and influence beneficial product microbiota such as lactobacilli at the expense of more noxious spoilage-formers such as pseudomonads in order to not only protect product quality but also to better control dangerous pathogens. An important unexpoilted area that deserves further attention is the optimization of MAP parameters for bulk packaging and transportation of seafood products to distant markets.

## Figures and Tables

**Table 1:** Selected studies published from 2000 to 2016 reporting on the application of modified atmospheres for shelf life extension of fish and fishery products.

Product Type	Storage Temperature (^o^C)	Gas (CO_2_:N_2_:O_2_)	Ratio G:P	Storage Time (Days)	Source	Study Highlights
Bass (gutted)	3	70:30:0	2:1	9	[17]	50:20:30 preserved best microbial and sensorial quality for 7–9 days of shelf life
		70:10:20				
		60:10:30				
		60:40				
		50:20:30				
		0:79:21				
Barramundi	8	100:0:0	2:1	20	[71]	100 and 75% CO_2_ had significantly less biogenic amines than other treatments
		75:25:0				
		50:50:0				
		25:75:0				
		Air				
Bonito (salted)	2	30:60:10		31	[72]	All treatments < 7log CFU after 31 days, * significantly less bacteria at day 31
		65:35:0 *				
		80:20:0				
		Vacuum				
		Air				
Carp (portions)	4	30:70:0 20:0:80		10	[73]	6 days shelf life
						8 days shelf life
						3 days shelf life
Cod, Atlantic (de-salted fillets)	1	75:20:5		21	[30]	Up to 14 additional days shelf life with MAP alone. Combination of MAP and citric or sorbate adds up to 4 or 10 days, respectively
		Air				
Cod	2	60:40:0	2:1	17	[22]	Effects of chilling prior to MAP.
Cod	2	40:40:20	2:1	21	[74]	Effects of chilling in MAP or Air prior to Frozen Storage and subsequent thawed shelf life
Cod	2	40:40:20	2:1	21	[75]	Effects of long term frozen storage. Shelf life 14 days after thawing
Cod, Atlantic	0	Optimization study	2:1	14	[21]	37:0:63 optimal for bacterial growth limit coupled with exudate min
Cod, Atlantic	1.3	60:40		21	[66]	Use of CO_2_ emitter without emitter
Cod, Atlantic	0	50:50:0	2:1	11	[76]	MAP w/O_2_ judged best, 16 days shelf life
		50:0:50				
		Air				
Cod, Atlantic	1.5, −0.9	50:45:5		21	[6]	Superchilled and MAP synergistic, 21 days or more shelf life
		Air				
Cod, Atlantic (loins)	−2	50:45:5	2 to 1		[7]	Brining of loins reduced shelf life, 21 days or more shelf life
Cod, Atlantic (brined)	0, −2, −3.6	50:45:5		28	[77]	Superchilled storage, 28 days or longer by TPC
		Air				
Cod, Atlantic	1.7	40:0:60	2:1	15	[78]	Transportation method effects
		Air				
Crayfish	2	80:10:10		21	[79]	21 days shelf life by APC
		PVC film				
		Vacuum				
Crayfish	2	80:10:10		10	[29]	MAP promoted activation of m-calpain and loss of calpastatin, but proteolysis did not correlate with texture
		PVC film				
		Vacuum				
Dolphinfish	1	45:50:5	2.5:1	18	[42]	AO = antioxidants (halophyte plant extract includes polyphenols: flavonoids, caffeic acid esters, coumarins)
		45:50:5 + AO				
Flounder (fillets)	4, 10	100:0:0	6:1	25	[80]	3000 OTR (oxygen transmission rate) at 4 °C, no C. bot toxin production up to 25 days but spoilage on day 15, at 10 °C sensory rejection on day 5 and toxin formation on day 8. VP: spoilage on day 8–9 at 10 °C, and toxic on day 20 at 4 °C
		VP				
		3000 OTR				
Hake (whole gutted)	2	60:25:15 (C)		30	[38]	modified (M) or controlled (°C) atmosphere packaging. Controlled more effective. Suggest 40% CO_2_ for least flavor issues
		60:25:15 (M)				
		80:0:20 (C)				
		80:0:20 (M)				
		40:20:40 (C)				
		40:20:40 (M)				
		Air				
Halibut (fillets)	4	50:50:0	2:1	23	[81]	Shelf life 20 days, MAP w/O_2_ best
		50:0:50				
		Air				
Lingcod	2	60:60:0 + CHI *		21	[82]	TPC shelf life 21 days for all but Air, * CHI:Chitosan, ** krill oil
		MAP + CHI + EO **				
		Vacuum + CHI				
		Vacuum + CHI + EO				
		Air				
Lobster, Norway	2 6	*		5 + 3 (8 total)	[83]	* 20 gas mixtures evaluated 80:10:10 = 13 days shelf life
*L. japonicus*	−1.5	40:30:30 + SC *		16	[8]	* superchill
		MAP				
Mackerel	5	30:65:5		12	[84]	min 60% CO_2_ for inhibitory effect on biogenic amines
		60:35:5				
		80:15:5				
		100:0:0				
		Vacuum				
		Air				
Mackerel	6	40:50:2	2:1	7	[39]	5 days shelf life
Mackerel, Atlantic horse	2, 4, 6, 10	48:50:2	2:1	11	[3]	7 days shelf life at 2C
Mullet, red striped	1	50:50:0 *	2.5:1	24	[85]	* Pre-treatment with ozone 10 days shelf life
		50:50:0				
		Air				
Mussels	2	50:50:0	3:1	12	[86]	65 & 80% CO_2_ longest shelf life at 8 days
		80:20:0				
		65:35:0				
		Air				
		Vacuum				
Mussels	4	50:50:0		15	[87]	Shelf life Air 7 days, MAP 11 days by APC; Air 11 days, MAP 13 days by sensory
		100:0:0				
		Air				
Prawns	0	50:50:0		10	[88]	MAP increased shelf life by 40 h
		Air				
Prawns	4	60:40:0		16	[35]	MAP controls black spot
Octopus, cooked	3, 24	CO_2_ *	4:1	28	[89]	* CO_2_ solubilized into product prior to packaging
		Vacuum				
Salad, marinated seafood	2	70:30:0	2:1	7 months	[90]	Sensory shelf life 7 months MAP, 4 months Air, TPC acceptable entire study
		50:50:0				
		Air				
Salmon, Atlantic (fillets)	2	60:40:0 *		21	[91]	Effect of freezing at −20 and −30 °C prior to MAP.
Salmon, Atlantic	2	90:10:0	1.2, 2.2, 2.5	28	[40]	90% CO_2_ 22 days shelf life Evaluated various barriers and additives
		75:25:0				
		60:40:0				
		40:60:0				
		25:75:0				
Salmon, Atlantic	−2	60:40:0		24	[65]	*Superchill* vs. Chill 24 days SC + MAP, <1000 cfu/g
		Air				
Salmon, Atlantic (fillets pre-rigor)	0.1	60:40:0		28	[41]	*Superchill* vs. Chill Shelf life 28 days superchill w/MAP
		Air				
Salmon, Atlantic	2	90:10:0 to 60:40:0	2.5 to 1 and 1.2 to 1	23	[40]	Modeling study with superchilling
Salmon, Atlantic	0.3	60:40:0	3:1	22	[24]	Effect of crowding stress/pre-rigor packaging/Longest shelf life with low stress, 15 days
Salmon, Atlantic	2 (2 days) then 20 (2 h) then 8	50:50:0		10	[92]	*P. Phosphoreum* and *L. piscium* dominate
		Vacuum				
Salmon, Atlantic *	4	55:45:0		15	[51]	* Australian raised Shewanella and Carnobacterium predominate
		30:70:0		12		
Salmon, Atlantic	8	50:50:0		12	[52]	
Salmon, Atlantic	0, 10	98:2:0	3:1		[9]	Develop model to predict rate of spoilage
		55:45:0				
		30:70:0				
Sardine	4	60:40:0	2:1	15	[32]	Shelf life 12 days MAP
		Vacuum				
		Air				
Scallops	0, 6, 20	30:10:60	0.5:1, 1:1, 1.5:1, 2:1, 3:1	modeled	[93]	Optimal at 0 °C, 60:10:30, at 2:1 g:p = shelf life 21 days
		45:10:45				
		60:10:30				
		75:10:15				
Sea bass	4	80:10:10	3:1	18	[94]	Protection of protein functionality
Sea bass	2	40:60:0	1.5:1	8	[26]	MAP extended shelf life by 1 day
		Air				
Sea bass	4	40:60:0	3:1	21	[33]	11 days shelf life
		50:50:0				14 days shelf life
		60:40:0				14 days shelf life
		Air				4 days shelf life
Seabream	4	40:30:30	2:1	33	[95]	MAP combined with 0.8%, 0.4% oregano or light salting extends shelf life >17, 17, and 12 days, respectively
		Air				
Seabream, Gilthead	0, 5, 10, 15	20:0:80 *			[19]	* Used Air instead of O_2_ Models predict shelf life at 7, 8, 12, and 32 days for 5 °C with 0, 20, 50, 80% CO_2_, respectively
		50:0:50 *				
		80:0:20 *				
Seabream, Gilthead	0	50:0:50 *		48	[96]	* Used Air instead of O_2_ Treatment with nisin osmotic solution (20,000 IU/100 g)
Seabream, Gilthead	2, 4, 8	60:40:0		13	[97]	MAP effect on QIM
		60:10:30				
Seabream, Gilthead *	4	40:30:30		28	[98]	Not treated or treated w/alcohol + lactic acid, Chitosan, grapefruit seed extract, or thymol
		95:0:5				
Seer fish	0–2	70:0:30		32	[99]	Treatments include Sodium Acetate
		Air				
Shrimp	8, 12, 16	59.5:39.5:1	100:1	>20	[100]	Thymol and MAP inhibition of *Salmonella* synergistic
		Air				
Shrimp, brown (cooked in package)	2	35:50:15		50	[101]	39 days shelf life w/MAP by sensory
		Vacuum				
		Air				
Shrimp, brown	4	40:60:0	3:1	7	[102]	H_2_S producers higher in summer
Shrimp, Chinese	2	40:30:30 *		21	[103]	* 13 days shelf life
		100:0:0 **				** 17 days shelf life
		Air				Treatment evaluation includes ozone and bactericides
Shrimp, gray (brown)	4	0:100:0	2:1	12	[31]	Presence of O_2_ had inhibitory effect on TMA and ethanol production and some sulfides. Recommend MAP with high CO_2_ to inhibit bacteria and O_2_ to inhibit undesirable volatiles
		0:90:10				
		0:70:30				
		0:50:50				
		50:50:0				
		50:40:10				
		50:20:30				
		50:0:50				
Shrimp, pink	Ice 1.6	---	1:2	9	[104]	MAP 9 days shelf life
		Air				
		40:30:30				
		45:50:5				
Shrimp, pink	−17	0:100:0		365	[105]	Light did not impact oxidation, less tough, better color
Shrimp, pink	2, 5, 8	50:30:20	4:1	65+	[106]	Lm growth in MA packaged, 2 °C, 20–21 days max
Shrimp, pink	7, 15	40:60:0	3:1	25+	[107]	Brine treatments evaluated include lactic, acetic, citric, benzoic, sorbic acids. Evaluates growth of specific spoilage and pathogenic bacteria
Shrimp, red	−18	0:100:0	2.25:1		[108]	Melanosis inhibited w/MAP
		50:50:0				
		Vacuum				
		Sulfite				
Shrimp, tropical	8	50:50:0		32	[109]	*C. maltaromaticum* and *S. baltica* major spoilage organisms
Shrimp, white	4	80:10:10	3:1		[110]	MAP in combination with hexylresorcinol shelf life 12 days
		Air				
Shrimp, white	4	50:45:5	3:1	10	[111]	* Green Tea Extract, ** AS: Ascorbic acid MAP not able to inhibit lactic acid bacteria or melanosis
		MAP + GTE *				
		MAP + GTE + AS **				
		Air				
Shrimp, white	?	60:22:18 *	3:1	10	[112]	* bi-sulfite wash Shelf life 10 days MAP
		36:64:0 *				
		Air *				
		Air				
Shrimp, white	4	40:55:5		10	[113]	MAP treatment could retard break-down of the structure protein.
		60:35:5				
		80:15:5				
		Air				
Shrimp, white	4	80:15:5	3:1	10	[114]	Lower O_2_ helps with melanosis
		80:10:10				
		80:0:20				
		Air				
Shrimp, white		80:10:10	3:1	10	[115]	*S. putrefaciens* has role in melanosis
Shrimp, white	4	80:10:10 *	3:1	12	[116]	* Quercetin, cinnamic acid, 4-hexylresorcinol treatment, AO’s help with melanosis, not bacteria
		Air *				
Shrimp, white	−18	40:50:10		100	[117]	w/electrolyzed water treatment decreased bacteria but neg effect on volatile flavor
		30:50:20				
		Air				
Squid	2	20:80:0	2.5:1	10	[118]	10 days shelf life with 70% CO_2_
		50:50:0				
		70:30:0				
		Vacuum				
Tilapia (fillets)	0–2	Vacuum		20	[28]	Products pre-treated with acetic acid (1%)
		60:40		7		
Trout, Rainbow (fillets)	0–2	50:40:10		16–20	[119]	Substitution of nitrogen for argon did not improve quality or shelf life
		50:30:20				
		50:20:30				
Trout, Rainbow	2	40:60:0		26	[120]	Ar instead of N, less reduction in pH, color
		40:60 (Ar):0				
		Air				
Trout, Rainbow (fillets)		50:50:0	2:1	18	[121]	MAP delayed Lm growth
		20:0:80				
		90:7.5:2.5				
		Air				
		Vacuum				
Trout, Rainbow (brined)	4	45:50:5		21	[122]	13–14 days shelf life w/o EO + 7–8 days w/EO * Essential Oil, oregano
		MAP + 0.2% EO *				
		MAP + 0.4% EO				
Trout, Rainbow (cooked)	4	60:40:0		27	[123]	27 days shelf life MAP
		40:60:0				
		Air				
Trout, Rainbow	4	80:20:0		22	[34]	Treatments w/UV-C radiation did not extend shelf life, higher oxidation as well
		Air				
		Vacuum				
Tuna	0, 2, 5, 10, 20	Varying *	3:1	25	[124]	* CO_2_:N_2_ varied.
Tuna, Bluefin	3	40:60:0	2.5:1	18	[18]	Treatment with α-tocopherol (0.5% *w*/*w*) for color retention
		0:100:0				
		Air				
Tuna, Yellowtail	ice	100:0:0 *	7	3:1	[125]	* w/oxygen absorber, ^1^ CO_2_ generator; MAP protected natural antioxidant enzymes in the muscle
		100:0:0 *^,1^				
		Air *				

## Abbreviations

AO	Antioxidant
MA	Modified Atmosphere
MAP	Modified Atmosphere Packaging
CAP	Controlled Atmosphere Packaging
DF	Degree of Filling
SSOs	Specific Spoilage Organisms
G:P	gas:product ratio
C	Controlled
M	Modified
CHI	Chitosan
SC	Superchill
EO	Essential Oil
OTR	Oxygen Transmission Rate
PVC	Polyvinyl chloride
QIM	Quality Index method
TVB-N	Total Volatile Basic Nitrogen
TMA	Trimethylamine
TMAO	Trimethylamine Oxide
TVC	Total Viable Counts
TBA	Thiobarbituric Acid Values
TBARS	Thiobarbituric Acid Reactive Substances

## References

[1] Farber J.M. (1991). Microbiological aspects of modified-atmosphere packaging technology—A review. J. Food Prot..

[2] Food and Agriculture Organization (FAO) (2015). Post-harvest Changes in Fish. http://www.fao.org/fishery/topic/12320/en.

[3] Alfaro B., Hernández I., Baliño-Zuazo L., Barranco A. (2013). Quality changes of Atlantic horse mackerel fillets (*Trachurus trachurus*) packed in a modified atmosphere at different storage temperatures. J. Sci. Food Agric..

[4] Alfaro B., Hernández I., Le Marc Y., Pin C. (2013). Modelling the effect of the temperature and carbon dioxide on the growth of spoilage bacteria in packed fish products. Food Control.

[5] Sivertsvik M., Jeksrud W.K., Rosnes J.T. (2002). A review of modified atmosphere packaging of fish and fishery products—Significance of microbial growth, activities and safety. Int. J. Food Sci. Technol..

[6] Wang T., Sveinsdóttir K., Magnússon H., Martinsdóttir E. (2008). Combined application of modified atmosphere packaging and superchilled storage to extend the shelf life of fresh cod (*Gadus morhua*) loins. J. Food Sci..

[7] Lauzon H.L., Magnússon H., Sveinsdóttir K., Gudjónsdóttir M., Martinsdóttir E. (2009). Effect of brining, modified atmosphere packaging, and superchilling on the shelf life of cod (*Gadus morhua*) loins. J. Food Sci..

[8] Liu S.L., Lu F., Xu X.B., Ding Y.T. (2010). Super-chilling maintains freshness of modified atmosphere-packaged *Lateolabrax japonicus*. Int. J. Food Sci. Technol..

[9] Powell S.M., Ratkowsky D.A., Tamplin M.L. (2015). Predictive model for the growth of spoilage bacteria on modified atmosphere packaged Atlantic salmon produced in Australia. Food Microbiol..

[10] McMillin K.W. (2008). Where is MAP going? A review and future potential of modified atmosphere packaging for meat. Meat Sci..

[11] Arvanitoyannis I.S., Stratakos A.C. (2012). Application of modified atmosphere packaging and active/smart technologies to red meat and poultry: A review. Food Bioprocess Technol..

[12] Alonso V., Provincial L., Gil M., Guillén E., Roncalés P., Beltrán J.A. (2012). The impact of short-term feeding of magnesium supplements on the quality of pork packaged in modified atmosphere. Meat Sci..

[13] Food and Agriculture Organization (FAO) (2016). Processing Parameters Needed to Control Pathogens in Cold Smoked Fish. Chapter III. Potential Hazards in Cold-Smoked Fish: *Clostridium botulinum* Type E. http://www.fda.gov/Food/FoodScienceResearch/SafePracticesforFoodProcesses/ucm099239.htm.

[14] Stammen K., Gerdes D., Caporaso F., Martin R.E. (1990). Modified atmosphere packaging of seafood. Crit. Rev. Food Sci. Nutr..

[15] Gopal T.K.S., Ravishankar C.N. (2005). Modified Atmosphere Packaging of Fish—A Review. Fish. Technol..

[16] Kropf D.H., Mancini R.A., Dikeman M., Devine C. (2014). Packaging: Modified and controlled atmosphere. Encyclopedia of Meat Sciences.

[17] Torrieri E., Cavella S., Villani F., Masi P. (2006). Influence of modified atmosphere packaging on the chilled shelf life of gutted farmed bass (*Dicentrarchus labrax*). J. Food Eng..

[18] Torrieri E., Carlino P.A., Cavella S., Fogliano V., Attianese I., Buonocore G.G., Masi P. (2011). Effect of modified atmosphere and active packaging on the shelf-life of fresh bluefin tuna fillets. J. Food Eng..

[19] Tsironi T., Tsevdou M., Velliou E., Taoukis P. Modelling the effect of temperature and CO_2_ on microbial spoilage of chilled gilthead seabream fillets. Proceedings of the IV International Symposium on Applications of Modelling as an Innovative Technology in the Agri-Food-Chain: Model-IT 802.

[20] Davis H.K., Blackistone B.A. (2009). Fish and shellfish. Principles and Applications of Modified Atmosphere Packaging of Foods.

[21] Sivertsvik M. (2007). The optimized modified atmosphere for packaging of pre-rigor filleted farmed cod (*Gadus morhua*) is 63 mL/100 mL oxygen and 37 mL/100 mL carbon dioxide. LWT-Food Sci. Technol..

[22] Bøknæs N., Ǿsterberg C., Nielsen J., Dalgaard P. (2000). Influence of freshness and frozen storage temperature on quality of thawed cod fillets stored in modified atmosphere packaging. LWT-Food Sci. Technol..

[23] Himelbloom B.H., Oliveira A.C.M., Chantarachoti J., Crapo C.A. (2013). Ethanol development in tissues of spoiling whole pink salmon (*Oncorhynchus gorbuscha*). J. Food Qual..

[24] Hansen A.A., Rødbotten M., Eie T., Lea P., Rudi K., Mørkøre T. (2012). The effect of crowding stress on bacterial growth and sensory properties of chilled Atlantic salmon fillets. J. Food Sci..

[25] Fletcher G.C., Summers G., Corrigan V.K., Johanson M.R., Hedderley D. (2004). Optimizing gas mixtures for modified atmosphere packaging of fresh king salmon (*Oncorhynchus tshawytscha*). J. Aquat. Food Prod. Technol..

[26] Poli B.M., Messini A., Parisi G., Scappini F., Vigiani V., Giorgi G., Vincenzini M. (2006). Sensory, physical, chemical and microbiological changes in European sea bass (*Dicentrarchus labrax*) fillets packed under modified atmosphere/air or prepared from whole fish stored in ice. Int. J. Food Sci. Technol..

[27] Randell K., Hattula T., Skyttä E., Sivertsvik M., Bergslien H., Ahvenainen R. (1999). Quality of filleted salmon in various retail packages. J. Food Qual..

[28] Soccol M.C.H., Oetterer M., Gallo C.R., Spoto M.H.F., Biato D.O. (2005). Effects of modified atmosphere and vacuum on the shelf life of tilapia (*Oreochromis niloticus*) fillets. Braz. J. Food Technol..

[29] Chen G., Guttmann R.P., Xiong Y.L., Webster C.D., Romaire R.P. (2008). Protease activity in post-mortem red swamp crayfish (*Procambarus clarkii*) muscle stored in modified atmosphere packaging. J. Agric. Food Chem..

[30] Magnússon H., Sveinsdóttir K., Lauzon H.L., Thorkelsdóttir Á., Martinsdóttir E. (2006). Keeping quality of desalted cod fillets in consumer packs. J. Food Sci..

[31] Noseda B., Goethals J., De Smedt L., Dewulf J., Samapundo S., Van Langenhove H., Devlieghere F. (2012). Effect of O_2_:CO_2_ enriched atmospheres on microbiological growth and volatile metabolite production in packaged cooked peeled gray shrimp (*Crangon crangon*). Intl. J. Food Microbiol..

[32] Özogul F., Polat A., Özogul Y. (2004). The effects of modified atmosphere packaging and vacuum packaging on chemical, sensory and microbiological changes of sardines (*Sardina pilchardus*). Food Chem..

[33] Provincial L., Gil M., Guillén E., Alonso V., Roncalés P., Beltrán J.A. (2010). Effect of modified atmosphere packaging using different CO_2_ and N_2_ combinations on physical, chemical, microbiological and sensory changes of fresh sea bass (*Dicentrarchus labrax*) fillets. Int. J. Food Sci. Technol..

[34] Rodrigues B.L., da Silveira Alvares T., Sampaio G.S.L., Cabral C.C., Araujo J.V.A., Franco R.M., Junior C.A.C. (2016). Influence of vacuum and modified atmosphere packaging in combination with UV-C radiation on the shelf life of rainbow trout (*Oncorhynchus mykiss*) fillets. Food Control.

[35] Slattery S.L., Palmer P.J. (2014). Modified atmosphere packaging (MAP) for control of black spot formation in chilled prawns. J. Aquat. Food Prod. Technol..

[36] Blackistone B., Blackistone B.A. (2009). Principles and applications of MAP of foods. Principles and Applications of Modified Atmosphere Packaging of Foods.

[37] Ruiz-Capillas C., Moral A. (2001). Residual effect of CO_2_ on hake (*Merluccius merluccius* L.) stored in modified and controlled atmospheres. Eur. Food Res. Technol..

[38] Alfaro B., Hernandez I. (2013). Evolution of the indigenous microbiota in modified atmosphere packaged Atlantic horse mackerel (*Trachurus trachurus*) identified by conventional and molecular methods. Int. J. Food Microbiol..

[39] Fernández K., Aspé E., Roeckel M. (2009). Shelf-life extension on fillets of Atlantic salmon (*Salmo salar*) using natural additives, superchilling and modified atmosphere packaging. Food Control.

[40] Hansen A.Å., Mørkøre T., Rudi K., Langsrud Ø., Eie T. (2009). The combined effect of superchilling and modified atmosphere packaging using CO_2_ emitter on quality during chilled storage of pre-rigor salmon fillets (*Salmo salar*). J. Sci. Food Agric..

[41] Messina C.M., Bono G., Renda G., La Barbera L., Santulli A. (2015). Effect of natural antioxidants and modified atmosphere packaging in preventing lipid oxidation and increasing the shelf-life of common dolphinfish (*Coryphaena hippurus*) fillets. LWT-Food Sci. Technol..

[42] Yesudhason P., Gopal T.K.S., Ravishankar C.N., Lalitha K.V., Kumar K.N.A. (2009). Effect of modified atmosphere packaging on chemical, textural, microbiological and sensory quality of seer fish (*Scomberomorus commerson*) steaks packaged in thermoformed trays at 0–2 °C. J. Food Proc. Preserv..

[43] Kolbe H.B. (1882). Antiseptische eigenschaften der kohlensäure. J. Prakt. Chem..

[44] Knoche W. (1980). Chemical reactions of CO_2_ in water. Biophysics and Physiology of Carbon Dioxide.

[45] Sivertsvik M., Jeksrud W.K., Vågane Å., Rosnes J.T. (2004). Solubility and absorption rate of carbon dioxide into non-respiring foods: Part 1: Development and validation of experimental apparatus using a manometric method. J. Food Eng..

[46] Sivertsvik M., Jensen J.S. (2005). Solubility and absorption rate of carbon dioxide into non-respiring foods. Part 3: Cooked meat products. J. Food Eng..

[47] Sivertsvik M., Rosnes J.T., Jeksrud W.K. (2004). Solubility and absorption rate of carbon dioxide into non-respiring foods: Part 2: Raw fish fillets. J. Food Eng..

[48] Rotabakk B.T., Lekang O.I., Sivertsvik M. (2007). Volumetric method to determine carbon dioxide solubility and absorption rate in foods packaged in flexible or semi rigid package. J. Food Eng..

[49] Dalgaard P., Gram L., Huss H.H. (1993). Spoilage and shelf-life of cod fillets packed in vacuum or modified atmospheres. Int. J. Food Microbiol..

[50] González-Rodríguez M.A.-N., Sanz J.-J., Santos J.-Á., Otero A., García-López M.A.-L. (2002). Numbers and types of microorganisms in vacuum-packed cold-smoked freshwater fish at the retail level. Int. J. Food Microbiol..

[51] Powell S.M., Tamplin M.L. (2012). Microbial communities on Australian modified atmosphere packaged Atlantic salmon. Food Microbiol..

[52] Macé S., Cardinal M., Jaffrès E., Cornet J., Lalanne V., Chevalier F., Pilet M.-F., Dousset X., Joffraud J.J. (2014). Evaluation of the spoilage potential of bacteria isolated from spoiled cooked whole tropical shrimp (*Penaeus vannamei*) stored under modified atmosphere packaging. Food Microbiol..

[53] Chaix E., Couvert O., Guillaume C., Gontard N., Guillard V. (2015). Predictive microbiology coupled with gas (O_2_/CO_2_) transfer in food/packaging systems: How to develop an efficient decision support tool for food packaging dimensioning. Comp. Rev. Food Sci. Food Saf..

[54] Huss H. (1995). Quality and Quality Changes in Fresh Fish.

[55] Nielsen J., Hyldig G., Larsen E. (2002). ‘Eating Quality’ of Fish—A Review. J. Aquat. Food Prod. Technol..

[56] Botta J.R., Botta J.R. (1995). Sensory evaluation: Freshness quality grading. Evaluation of Seafood Freshness and Quality.

[57] Olafsdóttir G., Martinsdóttir E., Oehlenschläger J., Dalgaard P., Jensen B., Undeland I., Nilsen H. (1997). Methods to evaluate fish freshness in research and industry. Trend. Food Sci. Technol..

[58] Sveinsdóttir K., Martinsdóttir E., Thorsdóttir F., Schelvis R., Kole A., Thorsdóttir I. (2010). Evaluation of farmed cod products by a trained sensory panel and consumers in different test settings. J. Sens. Stud..

[59] European Food Safety Authority (EFSA) (2015). Scientific and technical assistance on the evaluation of the temperature to be applied to pre-packed fishery products at retail level. EFSA J..

[60] Howgate P., Johnston A., Whittle K.J. (1992). Multilingual Guide to EC Freshness Grades for Fishery Products.

[61] Sea Fish Industry Authority (SFIA) (1984). Fish Purchase Specifications. http://www.seafish.org/media/Publications/specifications_for_the_purchase_of_fish_revised_03.pdf.

[62] QIM Eurofish. http://www.qim-eurofish.com.

[63] Howgate P. (2015). A history of the development of sensory methods for the evaluation of freshness of fish. J. Aquat. Food Prod. Technol..

[64] Svanevik C.S., Roiha I.S., Levsen A., Lunestad B.T. (2015). Microbiological assessment along the fish production chain of the Norwegian pelagic fisheries sector—Results from a spot sampling programme. Food Microbiol..

[65] Sivertsvik M., Rosnes J.T., Kleiberg G.H. (2003). Effect of modified atmosphere packaging and superchilled storage on the microbial and sensory quality of Atlantic salmon (*Salmo salar*) fillets. J. Food Sci..

[66] Hansen A.Å., Mørkøre T., Rudi K., Olsen E., Eie T. (2007). Quality changes during refrigerated storage of MA-packaged pre-rigor fillets of farmed Atlantic cod (*Gadus morhua* L.) using traditional MAP, CO_2_ emitter, and vacuum. J. Food Sci..

[67] Milne D., Powell S.M. (2014). Limited microbial growth in Atlantic salmon packed in a modified atmosphere. Food Control.

[68] Mørkøre T., Tahirovic V., Einen O. (2008). Impact of starvation and handling stress on rigor development and quality of Atlantic salmon (*Salmon salar* L.). Aquaculture.

[69] Kaale L.D., Eikevik T.M. (2014). The development of ice crystals in food products during the superchilling process and following storage, a review. Trends Food Sci. Technol..

[70] Duun A.S., Rustad T. (2007). Quality changes during superchilled storage of cod (*Gadus morhua*) fillets. Food Chem..

[71] Yassoralipour A., Bakar J., Rahman R.A., Bakar F.A. (2012). Biogenic amines formation in barramundi (*Lates calcarifer*) fillets at 8 °C kept in modified atmosphere packaging with varied CO_2_ concentration. LWT-Food Sci. Technol..

[72] Caglak E., Cakli S., Kilinc B. (2012). Effect of modified atmosphere packaging on quality and shelf life of salted bonito (*Sarda sarda*). J. Aquat. Food Prod. Technol..

[73] Hudecová K., Buchtová H., Steinhauserová I. (2010). Effects of modified atmosphere packaging on the microbiological properties of fresh common carp (*Cyprinus carpio* L.). Acta Vet. Brno.

[74] Bøknæs N., Østerberg C., Sørensen R., Nielsen J., Dalgaard P. (2001). Effects of technological parameters and fishing ground on quality attributes of thawed, chilled cod fillets stored in modified atmosphere packaging. LWT-Food Sci. Technol..

[75] Bøknæs N., Jensen K.N., Guldager H.S., Østerberg C., Nielsen J., Dalgaard P. (2002). Thawed chilled Barents Sea cod fillets in modified atmosphere packaging-application of multivariate data analysis to select key parameters in good manufacturing practice. LWT-Food Sci. Technol..

[76] Hovda M.B., Lunestad B.T., Sivertsvik M., Rosnes J.T. (2007). Characterization of the bacterial flora of modified atmosphere packaged farmed Atlantic cod (*Gadus morhua*) by PCR-DGGE of conserved 16S rRNA gene regions. Int. J. Food Microbiol..

[77] Gudjónsdóttir M., Lauzon H.L., Magnússon H., Sveinsdóttir K., Arason S., Martinsdóttir E., Rustad T. (2011). Low field nuclear magnetic resonance on the effect of salt and modified atmosphere packaging on cod (*Gadus morhua*) during superchilled storage. Food Res. Int..

[78] Hansen A.Å., Rødbotten M., Lea P., Rotabakk B.T., Birkeland S., Pettersen M.K. (2015). Effect of transport packaging and repackaging into modified atmosphere on shelf life and quality of thawed Atlantic cod loins. Packag. Technol. Sci..

[79] Chen G., Xiong Y.L. (2008). Shelf-stability enhancement of precooked red claw crayfish (*Cherax quadricarinatus*) tails by modified CO_2_/O_2_/N_2_ gas packaging. LWT-Food Sci. Technol..

[80] Arritt F.M., Eifert J.D., Jahncke M.L., Pierson M.D., Williams R.C. (2007). Effects of modified atmosphere packaging on toxin production by *Clostridium botulinum* in raw aquacultured summer flounder fillets (*Paralichthys dentatus*). J. Food Prot..

[81] Hovda M.B., Sivertsvik M., Lunestad B.T., Lorentzen G., Rosnes J.T. (2007). Characterization of the dominant bacterial population in modified atmosphere packaged farmed halibut (*Hippoglossus hippoglossus*) based on 16S rDNA-DGGE. Food Microbiol..

[82] Duan J., Jiang Y., Cherian G., Zhao Y. (2010). Effect of combined chitosan-krill oil coating and modified atmosphere packaging on the storability of cold-stored lingcod (*Ophiodon elongates*) fillets. Food Chem..

[83] Gornik S.G., Albalat A., Theethakaew C., Neil D.M. (2013). Shelf life extension of whole Norway lobster *Nephrops norvegicus* using modified atmosphere packaging. Int J. Food Microbiol..

[84] Yew C.C., Bakar F.A., Rahman R.A., Bakar J., Zaman M.Z., Velu S., Shariat M. (2014). Effects of modified atmosphere packaging with various carbon dioxide composition on biogenic amines formation in Indian mackerel (*Rastrelliger kanagurta*) stored at 5 ± 1 °C. Packag. Technol. Sci..

[85] Bono G., Badalucco C. (2012). Combining ozone and modified atmosphere packaging (MAP) to maximize shelf-life and quality of striped red mullet (*Mullus surmuletus*). LWT-Food Sci. Technol..

[86] Caglak E., Cakli S., Kilinc B. (2008). Microbiological, chemical and sensory assessment of mussels (*Mytilus galloprovincialis*) stored under modified atmosphere packaging. Eur. Food Res. Technol..

[87] Ulusoy Ş., Özden Ö. (2011). Preservation of stuffed mussels at 4 °C in modified atmosphere packaging. J. Aquat. Food Prod. Technol..

[88] Simoes J.S., Mársico E.T., Lázaro C.A., Ferreira M.D.S., Franco R.M., Pereira A.P.A., Conte-Junior C.A. (2015). Microbiological, physical and chemical characteristics of freshwater prawns (*Macrobrachium rosenbergii*) in modified-atmosphere packaging. Int. J. Food Sci. Technol..

[89] Mendes R., Silva H.A., Anacleto P., Cardoso C. (2011). Effect of CO_2_ dissolution on the shelf life of ready-to-eat Octopus vulgaris. Innov. Food Sci. Emerg. Technol..

[90] Gunsen U., Ozcan A., Aydin A. (2010). The Effect of modified atmosphere packaging on extending shelf-lifes of cold storage marinated seafood salad. J. Anim. Vet. Adv..

[91] Emborg J., Laursen B.G., Rathjen T., Dalgaard P. (2002). Microbial spoilage and formation of biogenic amines in fresh and thawed modified atmosphere-packed salmon (*Salmo salar*) at 2 °C. J. Appl. Microbiol..

[92] Macé S., Cornet J., Chevalier F., Cardinal M., Pilet M.F., Dousset X., Joffraud J.J. (2012). Characterisation of the spoilage microbiota in raw salmon (*Salmo salar*) steaks stored under vacuum or modified atmosphere packaging combining conventional methods and PCR–TTGE. Food Microbiol..

[93] Simpson R., Carevic E., Rojas S. (2007). Modelling a modified atmosphere packaging system for fresh scallops (*Argopecten purpuratus*). Packag. Technol. Sci..

[94] Masniyom P., Benjakul S., Visessanguan W. (2004). ATPase activity, surface hydrophobicity, sulfhydryl content and protein degradation in refrigerated seabass muscle in modified atmosphere packaging. J. Food Biochem..

[95] Goulas A.E., Kontominas M.G. (2007). Combined effect of light salting, modified atmosphere packaging and oregano essential oil on the shelf-life of sea bream (*Sparus aurata*): Biochemical and sensory attributes. Food Chem..

[96] Tsironi T.N., Taoukis P.S. (2010). Modeling microbial spoilage and quality of gilthead seabream fillets: Combined effect of osmotic pretreatment, modified atmosphere packaging, and nisin on shelf life. J. Food Sci..

[97] Campus M., Bonaglini E., Cappuccinelli R., Porcu M.C., Tonelli R., Roggio T. (2011). Effect of modified atmosphere packaging on Quality Index Method (QIM) scores of farmed gilthead seabream (*Sparus aurata* L.) at low and abused temperatures. J. Food Sci..

[98] Speranza B., Bevilacqua A., Conte A., Del Nobile M.A., Sinigaglia M., Corbo M.R. (2013). Use of desirability approach to predict the inhibition of *Pseudomonas fluorescens*, *Shewanella putrefaciens* and *Photobacterium phosphoreum* in fish fillets through natural antimicrobials and modified atmosphere packaging. Food Bioprocess Technol..

[99] Yesudhason P., Lalitha K.V., Gopal T.K.S., Ravishankar C.N. (2014). Retention of shelf like and microbial quality of seer fish stored in modified atmosphere packaging and sodium acetate pretreatment. Food Packag. Shelf Life.

[100] Zhou S., Sheen S., Pang Y.H., Liu L., Yam K.L. (2013). Antimicrobial effects of vapor phase thymol, modified atmosphere, and their combination against *Salmonella* spp. on raw shrimp. J. Food Sci..

[101] Kamadia V.V., Schilling M.W., Marshall D.L. (2013). Cooking and packaging methods affect consumer acceptability and shelf Life of ready-to-eat gulf brown shrimp. J. Aquat. Food Prod. Technol..

[102] Calliauw F., De Mulder T., Broekaert K., Vlaemynck G., Michiels C., Heyndrickx M. (2016). Assessment throughout a whole fishing year of the dominant microbiota of peeled brown shrimp (*Crangon crangon*) stored for 7 days under modified atmosphere packaging at 4 °C without preservatives. Food Microbiol..

[103] Lu S. (2009). Effects of bactericides and modified atmosphere packaging on shelf-life of Chinese shrimp (*Fenneropenaeus chinensis*). LWT-Food Sci. Technol..

[104] Goncalves A.C., López-Caballero M.E., Nunes M.L. (2003). Quality changes of deepwater pink shrimp (*Parapenaeus longirostris*) packed in modified atmosphere. J. Food Sci..

[105] Bak L.S., Andersen A.B., Andersen E.M., Bertelsen G. (1999). Effect of modified atmosphere packaging on oxidative changes in frozen stored cold water shrimp (*Pandalus borealis*). Food Chem..

[106] Mejlholm O., Bøknæs N., Dalgaard P. (2005). Shelf life and safety aspects of chilled cooked and peeled shrimps (*Pandalus borealis*) in modified atmosphere packaging. J. Appl. Microbiol..

[107] Mejlholm O., Devitt T.D., Dalgaard P. (2012). Effect of brine marination on survival and growth of spoilage and pathogenic bacteria during processing and subsequent storage of ready-to-eat shrimp (*Pandalus borealis*). Intl. J. Food Microbiol..

[108] Bono G., Badalucco C.V., Cusumano S., Palmegiano G.B. (2011). Toward shrimp without chemical additives: A combined freezing-MAP approach. LWT-Food Sci. Technol..

[109] Macé S., Joffraud J.J., Cardinal M., Malcheva M., Cornet J., Lalanne V., Chevalier F., Sérot T., Pilet M.-F., Dousset X. (2013). Evaluation of the spoilage potential of bacteria isolated from spoiled raw salmon (*Salmo salar*) fillets stored under modified atmosphere packaging. Int. J. Food Microbiol..

[110] Thepnuan R., Benjakul S., Visessanguan W. (2008). Effect of pyrophosphate and 4-hexylresorcinol pretreatment on quality of refrigerated white shrimp (*Litopenaeus vannamei*) kept under modified atmosphere packaging. J. Food Sci..

[111] Nirmal N.P., Benjakul S. (2011). Retardation of quality changes of Pacific white shrimp by green tea extract treatment and modified atmosphere packaging during refrigerated storage. Int. J. Food Microbiol..

[112] Kalleda R.K., Han I.Y., Toler J.E., Chen F., Kim H.J., Dawson P.L. (2013). Shelf life extension of shrimp (white) using modified atmosphere packaging. Pol. J. Food Nutr. Sci..

[113] Qian Y.F., Yang S.P., Xie J., Xiong Q., Gao Z.L. (2013). Impact of the O_2_ concentrations on bacterial communities and quality of modified atmosphere packaged Pacific white shrimp (*Litopenaeus Vannamei*). J. Food Sci..

[114] Qian Y.F., Xie J., Yang S.P., Wu W.H. (2013). Study of the quality changes and myofibrillar proteins of white shrimp (*Litopenaeus vannamei*) under modified atmosphere packaging with varying CO_2_ levels. Eur. Food Res. Technol..

[115] Qian Y.F., Xie J., Yang S.P., Wu W.H., Xiong Q., Gao Z.L. (2014). In vivo study of spoilage bacteria on polyphenoloxidase activity and melanosis of modified atmosphere packaged Pacific white shrimp. Food Chem..

[116] Qian Y.F., Xie J., Yang S.P., Huang S., Wu W.H., Li L. (2015). Inhibitory effect of a quercetin-based soaking formulation and modified atmospheric packaging (MAP) on muscle degradation of Pacific white shrimp (*Litopenaeus vannamei*). LWT-Food Sci. Technol..

[117] Zhang B., Ma L.K., Deng S.G., Xie C., Qiu X.H. (2015). Shelf-life of pacific white shrimp (*Litopenaeus vannamei*) as affected by weakly acidic electrolyzed water ice-glazing and modified atmosphere packaging. Food Control.

[118] Bouletis A.D., Arvanitoyannis I.S., Hadjichristodoulou C., Neofitou C., Sakkomitrou M., Kolokythopoulou F. (2014). The effect of modified atmosphere packaging on the microbiological, physical, chemical and sensory characteristics of broadtail squid (*Illex coindetii*). Int. J. Food Sci. Technol..

[119] Gimenez B., Roncales P., Beltran J.A. (2002). Modified atmosphere packaging of filleted rainbow trout. J. Sci. Food Agric..

[120] Choubert G., Brisbarre F., Parfouru D., Baccaunaud M. (2008). Argon modified atmosphere packaging for fillets of rainbow trout (*Oncorhynchus mykiss*) fed astaxanthin or canthaxanthin. J. Aquat. Food Prod. Technol..

[121] Yilmaz M., Ceylan Z.G., Kocaman M., Kaya M., Yilmaz H. (2009). The effect of vacuum and modified atmosphere packaging on growth of Listeria in rainbow trout (*Oncorhynchus mykiss*) fillets. J. Muscle Foods.

[122] Pyrgotou N., Giatrakou V., Ntzimani A., Savvaidis I.N. (2010). Quality assessment of salted, modified atmosphere packaged rainbow trout under treatment with oregano essential oil. J. Food Sci..

[123] Kaba N., Corapci B. (2014). Effects of two different modified atmosphere compositions on durability of steam-cooked rainbow trout (*Oncorhynchus Mykiss*, Walbaum, 1792). J. Food Proc. Preserv..

[124] Emborg J., Dalgaard P. (2008). Modelling the effect of temperature, carbon dioxide, water activity and pH on growth and histamine formation by *Morganella psychrotolerans*. Int. J. Food Microbiol..

[125] Tanimoto S., Song X.A., Sakaguchi M., Sugawara T., Hirata T. (2011). Levels of glutathione and related enzymes in yellowtail fish muscle subjected to ice storage in a modified atmosphere. J. Food Sci..

[126] Olafsdóttir G., Lauzon H.L., Martinsdóttir E., Oehlenschláuger J., Kristbergsson K. (2006). Evaluation of shelf life of superchilled cod (*Gadus morhua*) fillets and the influence of temperature fluctuations during storage on microbial and chemical quality indicators. J. Food Sci..

[127] Duun A.S., Rustad T. (2008). Quality of superchilled vacuum packed Atlantic salmon (*Salmo salar*) fillets stored at −1.4 and −3.6 °C. Food Chem..

[128] Kaale L.D., Eikevik T.M. (2015). The influence of superchilling storage methods on the location/distribution of ice crystals during storage of Atlantic salmon (*Salmo salar*). Food Control.

[129] Bahuaud D., Mørkøre T., Langsrud Ø., Sinnes K., Veiseth E., Ofstad R., Thomassen M.S. (2008). Effects of −1.5 °C super-chilling on quality of Atlantic salmon (*Salmo salar*) pre-rigor fillets: Cathepsin activity, muscle histology, texture and liquid leakage. Food Chem..

[130] Zeng Q.Z., Thorarinsdóttir K.A., Olafsdóttir G. (2005). Quality changes of shrimp (*Pandalus borealis*) stored under different cooling conditions. J. Food Sci..

[131] Barnett H.J., Stone F.E., Roberts G.C., Hunter P.J., Nelson R.W., Kwok J. (1982). Study in the use of a high concentration of CO_2_ in a modified atmosphere to preserve fresh salmon. Mar. Fish. Rev..

[132] Gram L., Dalgaard P. (2002). Fish spoilage bacteria–problems and solutions. Curr. Opin. Biotechnol..

[133] Li J., Liu Y., Li X., Zhu J., Fu L., Li T. (2010). Research advance in modified atmosphere packaging of aquatic products. J. Fish. Sci. China.

